# Supplementary material for: Identification of photoperiod-induced specific miRNAs in the adrenal glands of Sunite sheep (Ovis aries)
Source: Front Vet Sci. 2022 Jul 22;9:888207. doi: 10.3389/fvets.2022.888207 (PMC9354845; doi:10.3389/fvets.2022.888207)
Supplement: Supplementary file 1 [file Data_Sheet_1.ZIP › Supplementary file/miRDeep2 file.zip!/miRDeep2 file/Know_Structure/SP42A3/oar-mir-487a.pdf]

miRBase precursor : oar-mir-487a
Total read count : 56
oar-miR-487a-5p read count : 10
oar-miR-487a-3p read count : 46
remaining reads : 0

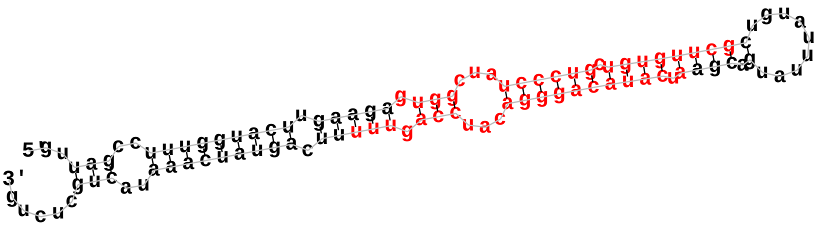

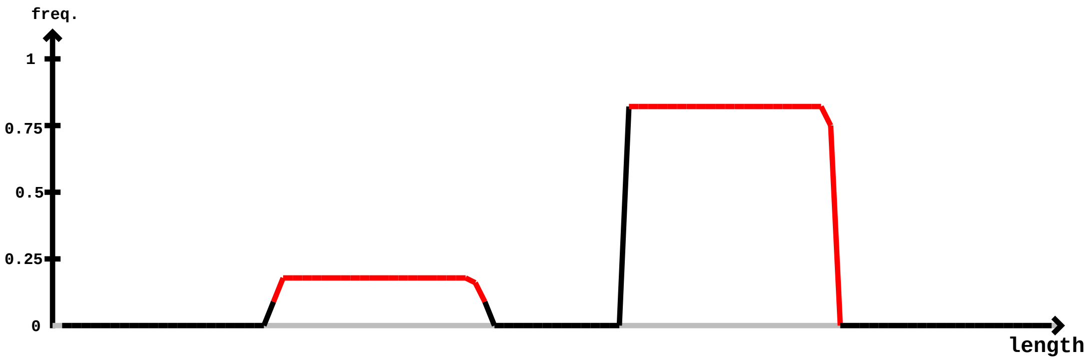


```
                                                                    oar-miR-487a-3p
                           oar-miR-487a-5p
5'- guuagccuuugguacuugaagaguggcuaucccugcuguguucgcuguauuuaugacgaaucauacagggacauccaguuuuucaguaucaaauacugcucug   -3'    exp
    ..(((..(((((((((.(((((.(((...((((((.(((((((((.........).)))).))))))))))...))).))))).)))))))))..))).....    reads     mm        sample
    .....................aguggcuaucccugcuguguu.............................................................    1         0         seq
    .....................aguggcuaucccugcuguguuc............................................................    3         0         seq
    .....................aguggcuaucccugcuguguucg...........................................................    1         0         seq
    ......................guggcuaucccugcuguguuc............................................................    1         0         seq
    ......................guggcuaucccugcuguguucg...........................................................    4         0         seq
    ..........................................................aaucauacagggacauccagu........................    4         0         seq
    ..........................................................aaucauacagggacauccaguu.......................    42        0         seq
```
